# Unpacking the black box

**DOI:** 10.31744/einstein_journal/2021ED6037

**Published:** 2020-12-17

**Authors:** Thiago Gonçalves dos Santos Martins, Paulo Schor

**Affiliations:** 1 Universidade de Coimbra Coimbra Portugal Universidade de Coimbra, Coimbra, Portugal.; 2 Universidade Federal de São Paulo São PauloSP Brazil Universidade Federal de São Paulo, São Paulo, SP, Brazil.

Today there are large databases (Big Data) of electronic records and digital images that allow pattern recognition in large volumes of information in a short period of time, contributing to the creation of a personalized Medicine with the aid of artificial intelligence.^(1,2)^

The algorithms used in medical diagnosis software are formed by a deep neural network, consisting of several layers that resemble the biological processes of the human cortex. Each neuron corresponds to a specific stimulus for a region within an image, similar to the way the brain neuron would respond to visual stimuli, activating a specific region of visual space. The neural network learns automatically by conferring values to these filters, even though specific parameters such as number of filters, filter size, and network architecture still need to be defined before the training phase.

The phase in which the device trains to improve its predictions is that of learning, which can be divided into two types: supervised and unsupervised. In supervised learning, information is assigned to the training data as they are entered into the computer, while in unsupervised learning, the device creates its own input algorithm. The training phase is followed by data validation, in which the figures should ideally be different from those used in the previous phase. Now the machine is able to classify the data and start making predictions. For this training phase, an algorithm needs thousands of data to reach acceptable accuracy.^(3,4)^

However, many researchers still cannot explain how the algorithms reached certain conclusions, and this slightly diminishes the confidence of the scientific world in the use of artificial intelligence in Medicine, since we tend to refute what we cannot explain. This term is known as “black box,” in which we do not have access to the information of the internal design and implementation of the algorithm. This term is opposed to “white box,” in which the component is completely exposed to the user. Among these algorithms, there is the “gray box,” which is when we only have access to some data.^(5)^

The most developed algorithms are formed by a non-linear structure with several layers in its architecture, enabling high complexity predictions. Nevertheless, this makes it difficult to explain how we get a result, which is easier in simpler and linear algorithms.^(6)^

Access to information of the algorithms is difficult, since it is not stored in a specific place, as is the human memory that - during learning - forms several synapses. Learning of the machines ends up forming these learning paths, attributing different weights to the filters during the training phase.

Unveiling the black box could create the possibility of seeing Medicine in a different way. Some animals, like the hummingbird, have a number and type of cones different from human beings, and develop the ability to identify colors imperceptible to our eyes, enabling them to see the world differently.^(7)^ Similarly, we can mention the function of algorithms, whose processing logic is not the same as that of human beings. From this point of view, even attesting to the results achieved by the algorithms, we are reluctant to trust the results since we do not understand the path taken. When we can explain the function of the algorithms, we can justify their decisions and minimize errors, thus making their vulnerability more evident. Learning with the machine can be exemplified with the development of programs such as AlphaGo, which allowed the teaching of new techniques of playing Go. This board game has been unveiled by machine learning, having a performance superior to that of humans.^(8)^In this way, by learning from the machines, we can expand our knowledge in other areas of science.

Some studies were carried out trying to explain the functioning of algorithms. Caruana et al.,^(9)^ described an algorithm for diagnosis of pneumonia and studied its behavior with cases of real patients. One must bear in mind that the algorithms created for the diagnosis and monitoring of diseases should preferably be validated with data from different populations, which were used in the training stage and with the participation of researchers who were independent, not taking part in development.^(10)^

Thus, the knowledge of how the human brain works can help us better understand the black box. A better understanding of machine learning can improve our ways of comprehending the world, enabling us to see Medicine with “hummingbird eyes”, and to begin to appreciate the mysteries of Medicine that, until then, have not been solved. In addition, it allows the development of better algorithms that need fewer examples for their learning.
