# Supplementary material for: Unpacking the black box
Source: Einstein (Sao Paulo). 2020 Dec 17;19:eED6037. doi: 10.31744/einstein_journal/2021ED6037 (PMC7793127; doi:10.31744/einstein_journal/2021ED6037)
Supplement: Translation

# Translation: Desembalando a caixa preta

**Authors:** Thiago Gonçalves dos Santos Martins, Paulo Schor

**Affiliations:** 1 Portugal Universidade de Coimbra, Coimbra, Portugal.; 2 Brasil Universidade Federal de São Paulo, São Paulo, SP, Brasil.

Na atualidade, existem grandes bancos de dados (*Big Data*) de prontuários eletrônicos e imagens digitais, que permitem o reconhecimento de padrões em grandes volumes de informações em curto período de tempo, contribuindo para a criação de uma Medicina personalizada com o auxílio da inteligência artificial.^(1,2)^

Os algoritmos utilizados nos programas de diagnóstico médico são formados por uma rede neural profunda, que consiste em diversas camadas que se assemelham aos processos biológicos do córtex humano, no qual cada neurônio responde a um estímulo específico para uma região dentro de uma imagem, semelhante à forma como o neurônio cerebral responderia aos estímulos visuais, que ativariam um região específica do espaço visual. A rede neural aprende sozinha, conferindo valores a esses filtros, embora os parâmetros específicos, como número de filtros, tamanho do filtro, arquitetura de rede, ainda precisem ser definidos antes da fase de treinamento.

A fase na qual o dispositivo treina para melhorar suas previsões é a de aprendizado, que pode ser dividida em dois tipos: supervisionado e não supervisionado. Na aprendizagem supervisionada, os dados são atribuídos aos dados de treinamento, conforme são inseridos no computador, enquanto no aprendizado não supervisionado, o dispositivo cria seu próprio algoritmo de entrada. A fase de treinamento é seguida pela de validação dos dados, que devem idealmente ser diferentes dos usados na fase anterior. Nessa fase, a máquina consegue classificar os dados e começar a realizar previsões. Para essa fase de treinamento, um algoritmo precisa de milhares de dados para ter uma acurácia aceitável.^(3,4)^

Contudo, muitos pesquisadores ainda não conseguem explicar como os algoritmos chegaram a certas conclusões, e isso diminui um pouco a confiança do mundo científico no uso da inteligência artificial na Medicina, já que tendemos a refutar o que não conseguimos explicar. Esse termo é conhecido como “*black box*”, na qual não temos acesso às informações do desenho interno e da implementação do algoritmo. Esse termo se opõe a “*white box*”, na qual o componente é completamente exposto ao usuário. Entre esses algoritmos, temos a “*gray box*”, que é quando temos acesso a alguns dados.^(5)^

Os algoritmos mais desenvolvidos são formados por uma estrutura não linear e com várias camadas em sua arquitetura, tornando possíveis previsões de alta complexidade. Contudo, isso dificulta a explicação de como chegamos a um resultado, o que é mais fácil em algoritmos mais simples e lineares.^(6)^

O acesso à informação dos algoritmos é difícil, já que esta não fica armazenada em um local específico, assim como a memória humana, que forma diversas sinapses durante o aprendizado. O aprendizado das máquinas acaba formando esses caminhos de aprendizado, fornecendo pesos diferentes aos filtros durante a fase de treinamento.

Desvendar a *black box* poderia criar a possibilidade de enxergar a Medicina de outra forma. Alguns animais, como o beija-flor, possuem um número e tipo de cones diferentes dos seres humanos, desenvolvendo a capacidade de identificar cores imperceptíveis ao nosso olho, o que permite que eles enxerguem o mundo de outra forma.^(7)^ Em analogia, podemos citar o funcionamento dos algoritmos, cuja lógica de processamento não e a mesma do ser humano. Sob essa ótica, mesmo atestando os resultados alcançados pelos algoritmos, por não entendermos o caminho percorrido, temos relutância em confiar nos resultados. Quando conseguimos explicar o funcionamento dos algoritmos, podemos justificar suas decisões e minimizar os erros, tornando a vulnerabilidade do mesmo mais perceptível. O aprendizado com a máquina pode ser exemplificado com o desenvolvimento de programas como o AlphaGo, que permitiu o ensino de novas técnicas de jogar *Go* . Esse jogo de tabuleiro conseguiu ser desvendado pelo aprendizado de máquina, tendo desempenho superior ao humano.^(8)^ Dessa forma, aprendendo com as maquinas podemos ampliar nossos conhecimentos em outras áreas da ciência.

Já foram realizados alguns estudos procurando explicar o funcionamento de algoritmos. Caruana et al.^(9)^ descreveram um algoritmo para diagnóstico de pneumonia e estudaram sua conduta com casos de pacientes reais. Lembrando que os algoritmos desenvolvidos para o diagnostico e acompanhamento de doenças devem ser preferencialmente validados com dados de populações diferentes que foram utilizados na etapa de treinamento e com a participação de pesquisadores independentes do seu desenvolvimento.^(10)^

Dessa forma, o conhecimento de como funciona o cérebro humano pode nos ajudar a entender melhor a *black box*, assim como o melhor entendimento do aprendizado da máquina pode aperfeiçoar nossas formas de entender o mundo, podendo enxergar a Medicina com “olhos de beija-flor”, e começar a compreender os mistérios da Medicina que, até então, não foram solucionados, além de possibilitar o desenvolvimento de melhores algoritmos que precisem de menos exemplos para seu aprendizado.
